# Are We There Yet? Recent Progress in the Molecular Diagnosis and Novel Antifungal Targeting of *Aspergillus fumigatus* and Invasive Aspergillosis

**DOI:** 10.1371/journal.ppat.1003642

**Published:** 2013-10-24

**Authors:** William J. Steinbach

**Affiliations:** 1 Department of Pediatrics, Division of Pediatric Infectious Diseases, Duke University Medical Center, Durham, North Carolina, United States of America; 2 Department of Molecular Genetics and Microbiology, Duke University Medical Center, Durham, North Carolina, United States of America; The University of North Carolina at Chapel Hill, United States of America

Are we there yet? Every parent has heard this inexorable question, but it is especially wrenching to hear it from a patient with a fatal illness. When someone, or someone's child, is diagnosed with invasive aspergillosis (IA) caused by *Aspergillus fumigatus*, the outcome has historically been bleak. In 2013, the field is erupting with exciting advances on multiple scientific fronts. But whether those research findings will improve clinical outcomes is still unclear—does your spouse, child, or neighbor now live or die with this disease? Medical breakthroughs over the last few decades surrounding cancer and transplantation, the main underlying conditions of those afflicted with IA, have been nothing short of astonishing. However, this revolution has also spawned a plethora of immunocompromised patients to inhale fungal spores and develop IA. For many patients with an absent immune system, the second leading cause of death, just behind cancer remission or transplant failure, is infection. The leading infectious cause of death is invasive fungal infections, and the most common mortal fungal infection is *A. fumigatus*. So have we sufficiently advanced scientifically for today's clinical needs in order to diagnose and treat IA—are we there yet?

*A. fumigatus* is both a beautiful and frustrating fungus. The beauty lies in the pathogen's ability to readily adapt its niche based on the host. The same fungus that lives in compost piles can shear pulmonary blood vessels in immunosuppressed patients, masquerade as an allergic trigger in an ectopic individual, or hide as a saprophyte and slowly sap the strength from an unsuspecting normal host. The frustration with this fungus is the current difficulty in early and accurate diagnosis, a limited number of effective antifungal treatments coupled with the growing emergence of antifungal resistance, and some persistent basic mechanistic questions surrounding its growth and regulation.

## What Is the Current Diagnostic Method for Invasive Aspergillosis?

**Figure 1:**
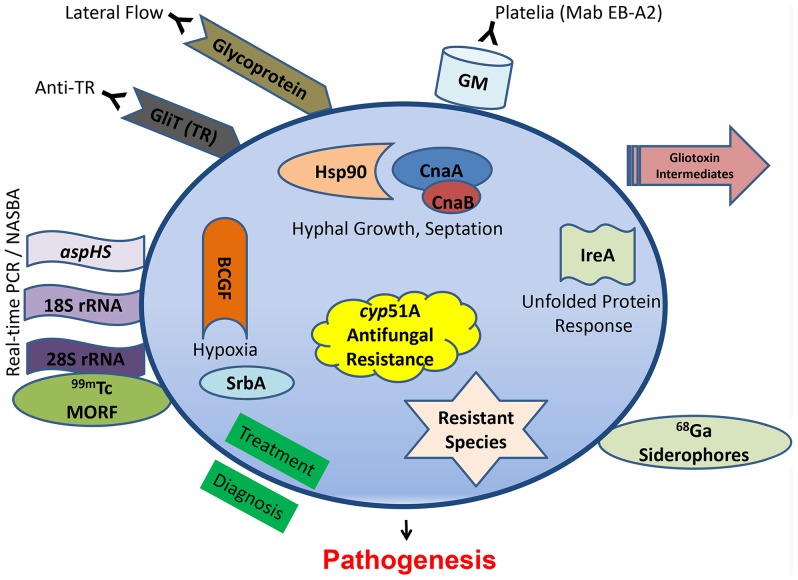
Recent diagnostic and therapeutic target advances against *Aspergillus fumigatus*. Diagnostic modalities are outside the circle, while novel antifungal targeting concepts, all shown to be significantly related to pathogenesis, are shown inside.

Clinically diagnosing invasive aspergillosis is often the most difficult of the common human pathogenic fungi triumvirate (*Candida*, *Cryptococcus*, and *Aspergillus*). For years, invasive aspergillosis remained recalcitrant to facile or proper diagnosis. Clinicians were relegated to ionizing radiation-laden CT scans to interpret small pixel changes, or dangerous biopsies in fragile patients. In May 2003, the US Food and Drug Administration approved the galactomannan assay (Platelia® *Aspergillus* EIA), a double-sandwich ELISA to detect galactomannan (GM) using a rat monoclonal antibody (Mab EB-A2) directed against tetra(1→5)-β-D-galactofuranoside, the immunodominant epitope of the *Aspergillus* cell wall antigen (Figure 1). This assay afforded the ability for noninvasive diagnosis, forever altering the diagnostic landscape. While GM has been validated as an effective tool, it is by no means a panacea to the diagnostic dilemma. It performs poorly in the setting of solid organ transplantation, use of mold-active antifungals drops its sensitivity, and there is cross-reactivity with certain beta-lactam antibiotics. More recently, testing bronchoalveolar lavage fluid with the GM assay reveals generally greater sensitivity and specificity than serum testing [1]. Another set of commercially available assays (Fungitell® in the United States) detect (1–3)-β-D-glucan in the cell wall of most fungi, including *Aspergillus*. Two consecutive positive assays have a high specificity for an invasive fungal infection, although the infecting fungal genus cannot be depicted, but sensitivity is lower and there are numerous cross-reactive concerns so results need to be carefully interpreted [2].

A newer antibody-based approach involves a lateral flow device, akin to a home pregnancy test, that offers ease of diagnosis and potential cost savings. First reported in 2008 [3], it is an immunochromotographic assay based on a mouse Mab that binds to a yet-unknown protein epitope present on an extracellular glycoprotein antigen. The assay is quite specific for *Aspergillus* spp., but a recent study in 101 patients showed inferior sensitivity to the GM assay [4]. Another antibody, found in the sera of non-neutropenic patients with invasive aspergillosis, reacted to the *A. fumigatus* secretory protein thioredoxin reductase GLiT (TR). Western analysis showed that the recombinant His_6_-tagged TR protein could be recognized in sera from infected animals and non-neutropenic patients via ELISA-detecting anti-TR antibodies [5], but unfortunately not earlier in the infection than the GM assay.

## Does PCR Improve Diagnosis?

PCR is an ultrasensitive diagnostic platform; however, PCR detection of *Aspergillus* remains limited by a lack of standardization, a problem undertaken by the European *Aspergillus* PCR Initiative (EAPCRI). Recently, the group tested 29 combinations of PCR variables used in 21 centers (showcasing the current wide variation), and excluded nested-PCR assays as too prone to false-positive results and unlikely to be used outside of referral centers [6]. Despite continued efforts, standardized and validated procedures have not been developed. A refinement to the *Aspergillus* PCR approach is real-time nucleic acid sequence–based amplification (NASBA), which can allow single-stranded RNA to be detected by molecular beacon probes [7]. This technology, targeting the 28S rRNA, offers robust sensitivity and specificity, but it is unclear if it can be translated to routine clinical laboratory practice. The commercially available Myconostica MycAssay *Aspergillus* PCR, targeting the 18S rRNA gene, is a real-time PCR with promise for detection of *Aspergillus* DNA in respiratory tract samples, but there are no reports yet in serum or blood [8]. Using a different genetic target, real-time PCR detection of *aspHS*, encoding for an *A. fumigatus*–secreted hemolysin, afforded detection in murine samples but unfortunately not consistently in clinical bronchoalveolar lavage samples due to inhibitors [9].

In general, PCR seems to be more sensitive than GM, but requires specialized equipment and laboratory technologists and a standardized protocol. Additionally, the potential PCR inhibitors present in clinical samples needs to be better addressed. The benefit of PCR is that while GM cannot identify infecting *Aspergillus* species, PCR could be tailored to the species level and also possibly infer general antifungal susceptibility patterns.

## What about Other Diagnostic Strategies?

There seems to be less recent focus on metabolomics, but infection with an overexpression strain for RsmA, a Yap-like basic leucine zipper protein found to regulate gliotoxin in *A. fumigatus*, reveals gliotoxin intermediates seen in murine lung infection [10]. Other new diagnostic approaches have focused on radiopharmaceutical imaging. The isotope carrier molecule Gallium (^68^Ga) radiolabeled to *A. fumigatus* siderophores demonstrated high and specific uptake by *A. fumigatus* using positron emission tomography (PET) imaging *in vitro* and in a rat model [11]. Iron plays an important role during infection and another imaging strategy used technetium (^99m^Tc)-labeled morpholino oligomers (MORF), DNA analogs in which the sugar is replaced by a morpholino moiety that binds to the complimentary DNA or RNA. Using 99mTc-labeled 28S rRNA, *Aspergillus* genus-specific and *A. fumigatus* species-specific probes confirmed the infectious biodistribution via single-photon emission computed tomography (SPECT) imaging of murine lungs [12]. These imaging-based diagnostic tools are useful for locating infection, or possible monitoring response to therapy, but their sensitivity in various patient populations and settings, as well as cross-reactivity with other pathogens, remains unclear.

## How Do We Improve Current Antifungal Treatment Options?

Antifungal discovery is tricky—it is complicated to design an agent to selectively kill one eukaryote (pathogen) while not harming the larger infected eukaryote (host). In pharmaceutical development lingo, treating *Aspergillus* is the gold standard. Designing a new antifungal with activity only against yeasts would be welcome, but without the ability to kill molds it would only be an incremental advance. Current guideline-recommended therapy against invasive aspergillosis is the triazole voriconazole, superior to the now generally antiquated polyene amphotericin B deoxycholate [13]. While a 2002 landmark clinical trial found an improved response with voriconazole (52.8%) versus amphotericin B (31.6%), the field is still in need of improved antifungal targets. Many felt the answer was a combination antifungal approach, akin to other medical disciplines where agents with different mechanisms are employed for synergistic response. This had been reported in *Aspergillus* with mixed results in *in vitro*, animal model, and small clinical studies. A recently completed multinational randomized clinical trial comparing voriconazole with and without the echinocandin anidulafungin showed no clear and definitive benefit, and only statistical trends in improvement in certain groups [14]. This is likely the end of that discussion, whereby currently available antifungal agents have now been tested and retested in combinations and show limited benefit. It is unlikely that the optimal therapeutic answer is an existing antifungal, or even a combination of existing possibilities; what is needed is an entirely new approach with a new target or pathway uncovered from solid basic pathogenesis studies. This is even more pressing with the increasing development of antifungal azole resistance amongst *A. fumigatus* isolates, generally through one of several *cyp*51A mutations [15], and also the emerging non-*fumigatus Aspergillus* species with antifungal resistance, such as *Aspergillus lentulus*, *Aspergillus calidoustus*, *Aspergillus udagawae*, *Neosartorya pseudofisheri*, and others [16].

## How Do We Target a More Promising Regulatory Pathway?

Decades of *Aspergillus* pathogenesis research have confirmed its multifactorial nature. There are myriad studies highlighting *A. fumigatus* virulence factors, all of which are possible antifungal drug targets and could not be reviewed here. Some of the more recent promising approaches target not specific putative virulence genes, but instead overarching pathways or regulatory circuits (Figure 1) for a more pleiotropic effect.

Calcineurin is a conserved protein phosphatase important in stress response, and deletions of multiple components of the pathway (*cnaA, cnaB, crzA, cbpA*, and *pmrA*) all lead to various growth and virulence defects. Recently, targeted mutations of fungal-specific calcineurin A residues, completely absent in humans, blocked phosphorylation and activation of this enzyme and led to a significant virulence defect [17]. Similarly, repression of calcineurin's molecular chaperone heat shock protein 90 (Hsp90) led to a substantial growth and virulence defect [18]. The unfolded protein response is also critical, and an Δ*ireA* strain, defective in the endoplasmic reticulum transmembrane sensor, is avirulent [19]. LaeA, which serves as a master regulator governing numerous aspects of secondary metabolite production, has also been shown to be involved in development, toxin production, and correlated to virulence in several *Aspergillus* species.

*A. fumigatus* invades and occludes blood vessels, causing tissue hypoxia. Basic fibroblast growth factor (BCGF) significantly potentiated the antifungal effects of amphotericin B through increasing neutrophil influx into infected murine tissue and reversed the antiangiogenic activity of *A. fumigatus* [20]. Angioinvasion is an important and understudied hallmark of *Aspergillus* pathogenesis, as its presence also prevents delivery of antifungals to the site of infection. Hypoxia adaptation itself has also been studied, and an Δ*srbA* mutant, defective in a sterol regulatory element–binding protein transcription factor, demonstrates the importance of this pathway for survival in the areas of inflammation [21].

Unfortunately, to move from “virulence factor” to drug target requires more than an impressive Kaplan-Meier curve and histology evidence. A “druggable” target is required, whereby molecules can be generated to target that entity. Therefore, the next critical steps for these exciting putative targets listed still remain. Similar to the future of molecular diagnosis, the therapeutic answer might be a new composite strategy—attacking known biosynthesis targets with adjunctive stress response inhibitors. While we are not there yet, we are moving faster than ever before to improve our diagnosis and treatment of invasive aspergillosis.
